# The *Alternaria* Mycotoxin Alternariol Triggers the Immune Response of IL‐1β‐stimulated, Differentiated Caco‐2 Cells

**DOI:** 10.1002/mnfr.201900341

**Published:** 2019-10-04

**Authors:** Cornelia Schmutz, Ebru Cenk, Doris Marko

**Affiliations:** ^1^ University of Vienna Faculty of Chemistry Department of Food Chemistry and Toxicology Waehringerstr. 38 A‐1090 Vienna Austria

**Keywords:** alternariol, cytokines, inflammation, microRNAs, mycotoxins

## Abstract

**Scope:**

Alternariol (AOH), a toxic secondary metabolite of *Alternaria spp*., may contaminate a broad spectrum of food and feed. Besides its cytotoxic, genotoxic, and estrogenic properties, several studies report the potential of AOH to suppress the rich network of immune responses. The specific effect of AOH on inflammation‐related signaling in non‐immune cells of the intestinal epithelial layer has, however, not been investigated yet. Since intestinal epithelial cells (IECs) are, compared to underlying cells, exposed to higher concentrations of the ingested mycotoxin, the question is addressed whether immunomodulation by AOH at the gastrointestinal barrier must be considered.

**Methods and results:**

The impact of AOH (0.02–40 µm) on inflammatory signaling in either IL‐1β‐stimulated or non‐stimulated differentiated Caco‐2 cells is determined. AOH significantly reduces IL‐1β transcription after 5 h but shows an increasing tendency on IL‐8 transcript levels after long‐term exposure (20 h). In IL‐1β‐stimulated cells, AOH (20–40 µm) augments TNF‐α transcripts while repressing IL‐8, IL‐6, and IL‐1β transcription as well as IL‐8 secretion. Furthermore, inflammation‐related microRNAs miR‐16, miR‐146a, miR‐125b, and miR‐155 are altered in response to AOH.

**Conclusion:**

The obtained data indicate that AOH represses immune responses in an inflamed environment, possibly leading to higher susceptibility to diseases.

## Graphical abstract

**Several toxic secondary metabolites of filamentous fungi**, so‐called mycotoxins, have the potential to modify immune responses. The results show that the food contaminant alternariol (AOH) alters the interleukin(IL)‐1β induced inflammatory response in non‐immune cells of the intestinal epithelium, most probably by targeting the NF‐*κ*B (nuclear factor *κ*B) pathway.

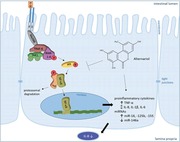

## 1 Introduction

**Figure 1:**
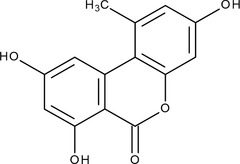
Chemical structure of alternariol (AOH).

Produced by filamentous fungi of *Alternaria* species, the mycotoxin alternariol (AOH, **Figure** 1) is an ubiquitously occurring contaminant of a broad variety of food and feed commodities. Spoilage through its main producing mold, *Alternaria alternata*, does not only occur during culture but also during cooled transportation and storage.^1^ Still lacking in occurrence and hazard data, the emerging mycotoxin AOH is not monitored and regulated yet. The European Food Safety Authority (EFSA) estimated the daily dietary intake of AOH to be low (1.0–15.2 ng kg^–1^ bodyweight (bw)), but nevertheless to exceed presumably the threshold of toxicological concern (TTC) of 2.5 ng kg^–1^ bw per day by far. A more detailed hazard characterization for AOH was, hence, demanded.^2^, ^3^

So far, AOH has been associated with a spectrum of toxic effects, ranging from moderate estrogenicity to cytotoxic,^1^ clastogenic,^4^ fetotoxic,^5^ and mutagenic effects, whereby genotoxicity has been attributed predominantly to its poisoning activity on topoisomerases.^6^ Furthermore, the induction of oxidative stress by AOH has been shown in several cell lines, including colon carcinoma cells Caco‐2,^7^ HT‐29,^8^ and murine macrophages RAW 264.7.^9^ Since the ability of a substance to induce oxidative stress is often linked to inflammation, the impact of AOH on inflammatory pathways has gained increasing interest in recent years. The morphology of human primary macrophages as well as RAW 264.7 cells, for instance, was found to be altered in response to AOH exposure.^10^ Additionally, Solhaug et al. reported impact on the expression of several cell surface markers during the phorbol 12‐myristate 13‐acetate (PMA) induced differentiation of THP‐1 monocytes to macrophages. In detail, the induction of both CD14 and CD11b was reduced by AOH treatment with concomitant induction of CD71 expression, that is usually decreased during the differentiation process.^11^ AOH was furthermore reported to not only interfere in inflammatory cytokine signaling in human primary macrophages, RAW 264.7, and differentiated THP‐1 macrophages, but also to reduce the lipopolysaccharide (LPS) induced immune response in THP‐1 as well as RAW 264.7 cells.^10^, ^11^, ^12^, ^13^ The observed immunosuppressive potential of AOH was very recently in part attributed to its potent inhibition of the LPS‐induced NF‐κB (nuclear factor kappa B) activation.^12^

Another cellular event that is potentially interfering in the posttranscriptional repression of cytokines is the modulation of short (18–24 nucleotides), noncoding RNAs called microRNAs (miRNAs). miRNAs were not only reported to tune and decrease translational efficiency, but also to destabilize or cleave target protein‐coding mRNA.^14^ Indeed, inflammation‐related miRNAs 155 and 146a were significantly induced and repressed, respectively, by AOH treatment (20 µm) in LPS stimulated, differentiated THP‐1 macrophages.^12^ The interpretation of the specific function of a miRNA, in general, is utterly complex as hundreds of targets can be regulated by a single miRNA. For the NF‐κB dependent miR‐146a two direct targets (TNF‐associated factor 6 (TRAF‐6) and IL‐1 receptor associated kinase (IRAK‐1)) that are both key adapter molecules in pathways mediated by TIRs (Toll‐like/IL‐1 receptors) were identified.^14^ Following AOH treatment, several studies reported reduced secretion of TNF‐α,^10^, ^12^, ^13^ which is—among others—a direct target of miR‐155.^15^

Since the main route of admission of mycotoxins is ingestion, the gastrointestinal tract (GI) often represents one of the target organs.^16^ It contains the body's biggest accumulation of immune cells, where a highly complex orchestration of interactions is needed to fight off ever‐invading food‐associated pathogens. The disturbance of any variable of this complex could induce a state of pathological or even chronic inflammation.^17^ Immunogenic functions are, however, not restricted to classic immune cells, such as dendritic cells or macrophages, as also intestinal epithelial cells (IECs) are able to exert them. Due to their location, lining the gastro‐intestinal tract, IECs are possibly facing higher concentrations of mycotoxins when compared to underlying cells of the mucosa. Additional to their function as physical barrier impeding the intrusion of pathogens, IECs aid in the promotion of immune responses by communicating with surrounding cells via the secretion of soluble, biologically active mediators.^18^ The impact of the xenobiotic AOH on inflammation‐related signaling in IECs has, however, not been addressed yet.

The present work, therefore, aimed to characterize the potential impact of the emerging mycotoxin AOH on intestinal inflammatory signaling. To elucidate possible effects on proinflammatory cytokines IL‐8, IL‐6, TNF‐α, and IL‐1β, differentiated Caco‐2 cells served as an in vitro model of the GI. Additionally, the role of AOH in regulating selected inflammation‐related miRNAs (125b, 155, 146a, and 16) was examined. All experiments were performed in either non‐stimulated cells or, mimicking conditions of an inflammatory situation, IL‐1β stimulated, differentiated Caco‐2 cells.

## 2 Experimental Section

### 2.1 Chemicals

The used standard of alternariol from *Alternaria* species (AOH) had a purity of ≈96 % and was purchased from Sigma–Aldrich (St. Louis, MO). To check for impurities with structural analogy LC–MS measurements according to Puntscher et al.^19^ were conducted and revealed the presence of 2.1–3.4% of alternariol monomethyl ether (AME). Stock solutions of AOH and dexamethasone (Dex, HPLC ≥ 98%; Sigma–Aldrich) were prepared in DMSO (Carl Roth GmbH&Co., Karlsruhe, Germany). Recombinant human IL‐1β (with human serum albumin) was purchased from InvivoGen (San Diego, CA) and diluted in endotoxin free water as instructed by the manufacturer.

### 2.2 Cell Culture and Differentiation

The epithelial Caco‐2 brushboarder‐expressing‐1 clone (C2BBe1 clone, ATCC CRL‐2102) was obtained from American Type Culture Collection (ATCC, Manassas, VA). Caco‐2 cells were cultured in DMEM containing 4.5 g L^−1^ glucose, supplemented with 1 mm sodium pyruvate, 0.01 mg mL^−1^ human transferrin, 10% heat inactivated fetal calf serum (FCS), and 1% penicillin/streptomycin (100 U mL^−1^/100 µg mL^−1^) at 37 °C and 5% CO_2_ in a humidified atmosphere. Medium and supplements were purchased from Thermo Fisher Scientific (Vienna, Austria). Cells were sub‐cultivated twice per week at 85% confluence, inoculating 1.0–1.5 × 10^6^ cells into flasks of 175 cm^2^ and never exceeding the cell passage number of 25. For characterization of the Caco‐2 cell monolayer that shall be used in experiments, cells were seeded into 12‐well Transwellplates (1.12 cm^2^ area, 0.4 µm membrane pore size; Sigma–Aldrich, St. Louis, MO) at a cell density of 85 000 cells cm^−2^ and during 21 days of cultivation the transepithelial electrical resistance (TEER) was measured with an EVOM^2^ voltohmmeter (World Precision Instruments, Sarasota, FL). In addition, the integrity of the monolayer was determined via a lucifer yellow permeability assay after 0, 7, and 21 days of cell cultivation. After 7 days of cultivation the TEER values were above 400 Ω cm^2^ and the integrity check had shown a permeability of ≈1 % (data not shown). Former studies obtained similar results, wherefore the used Caco‐2 cell monolayer can be considered as a representative in vitro GI cell model after 7 days of cultivation.^20^, ^21^, ^22^

### 2.3 Mycotoxin Treatment and Dosage Information

Caco‐2 cells were seeded at a cell density of 85 000 cells cm^−2^ and were cultivated for 7 days to obtain a tight and partially differentiated Caco‐2 cell monolayer before incubation with the test substance. During differentiation cell culture medium was changed three times per week.

Seven days post‐seeding medium was replaced with medium containing the test substances at required concentrations with a final DMSO concentration of 1%. Test concentrations and incubation times were chosen in accordance to a recent publication on immunomodulatory effects of AOH in THP‐1 macrophages.^12^ Briefly, cells were either incubated with AOH alone at concentrations of 0.02, 0.2, 2, and 20 µm for 5 or 20 h, or cells were first pre‐incubated for 2 h with the test compound and then additionally stimulated with IL‐1β (25 ng µL^−1^) for further 3 or 18 h. Additionally, a concentration of 40 µm AOH was applied on Caco‐2 cells, as the intestinal epithelial layer is exposed to higher concentrations of food‐associated contaminants in comparison to underlying cells of the lamina propria, e.g., macrophages. Intestinal inflammation was experimentally induced with the proinflammatory cytokine IL‐1β, which has served as inflammatory stimulus in various Caco‐2 studies,^23^, ^24^, ^25^ since these cells are to some extent unresponsive to LPS stimulation.^26^ In IL‐1β stimulated cells, the corticoid Dex served as a positive control for anti‐inflammatory effects, while it was co‐incubated with 40 µm AOH in non‐stimulated Caco‐2 cells to counteract a potential induction of inflammatory signaling by AOH.

### 2.4 Cell Viability Assay

Cell viability was assessed with the alamarBlue cell viability assay. After treating the Caco‐2 monolayer as described in Section 2.3, cell culture medium was removed and cells were washed with prewarmed PBS solution prior to incubation with resazurin in FCS free cell culture medium at a final concentration of 10% v/v. The Caco‐2 cell monolayer was incubated for 2 h with the alamarBlue reagent at 37 °C and 5% CO_2_ in the dark. During incubation the non‐fluorescent compound resazurin is absorbed by metabolically active cells and gets reduced in the cytosol to the fluorescent resorufin.^27^ After incubation, an aliquot of the cell culture medium was transferred into a 96‐well plate in triplicates and the fluorescence of resorufin was measured with the Gen5 Microplate Reader (BioTek, Vienna, Austria). To determine the fluorescence an excitation wavelength of 530 nm was used. The final read out of the emission was then performed at 560 nm.

### 2.5 Quantitative Real‐time PCR

To determine the mRNA transcript levels of IL‐1β, IL‐6, IL‐8, and TNF‐α as well as the miRNA transcript levels of miR‐16, miR‐125b, miR‐146a, and miR‐155, two‐step quantitative RT‐PCR (qRT‐PCR) was performed. Dexamethasone (1 µm), a corticosteroid, served as positive control and 1% v/v of DMSO as solvent control. Following incubation (see Section 2.3), cells were washed with ice‐cold PBS, lysed with Qiazol (Qiagen, Hilden, Germany) and total RNA was extracted using the miRNeasy Kit (RNA size ≥ 18 nucleotides, Qiagen) according to the instructions of the manufacturer's protocol. The purity and quantity of the obtained RNA extract was analyzed with the NanoDrop 2000 (Thermo Fisher Scientific). Afterward, total RNA was reverse transcribed into complementary DNA (cDNA) by using the miScript II RT Kit (Qiagen). The gene specific cDNA was exponentially amplified by carrying out qRT‐PCR using the StepOne Plus PCR System (Applied Biosystems, Thermo Fisher Scientific). qRT‐PCR was conducted in a 96‐well plate using the miScript SYBR Green PCR Kit (Qiagen) and gene‐specific‐ or miRNA‐specific primer assays (Qiagen) in a final reaction volume of 20 µL. Following primer assays were used: β‐Actin (Hs_ACTB_1_SG; QT00095431), glyceraldehyde 3‐phosphate dehydrogenase (GAPDH; Hs_GAPDH_1_SG; QT00079247), IL‐1β (Hs_IL1B_1_SG; QT00021385), IL‐6 (Hs_IL6_1_SG; QT00083720), IL‐8 (Hs_CXCL8_1_SG; QT00000322), TNF‐α (Hs_TNF_1_SG; QT00029162), U6 Small Nuclear 2 RNA (RNU6; Hs_RNU6‐2_11; MS00033740), small nucleolar RNA, C/D box 68 (SNORD68; Hs_SNORD68_11; MS00033712), miR‐16 (Hs_miR‐16_2; MS00031493), miR‐125b (Hs_miR‐125b_1; MS00006629), miR‐146a (Hs_miR‐146a_1; MS00003535), and miR‐155 (Hs_miR‐155_2; MS00031486). The amplification protocol started with an initial activation step of the HotStarTaq polymerase for 15 min at 95 °C, followed by 40 cycles of denaturation for 15 s at 94 °C, annealing for 30 s at 55 °C, and extension for 30 s at 70 °C, and a final melting curve analysis. Relative mRNA and miRNA transcript levels were calculated by using the 2^−ΔΔC^_T_ method as PCR efficiencies were comparable.^28^, ^29^ The obtained C_T_ values of the target genes and miRNAs were normalized to the average of the C_T_ values of the housekeeping genes (GAPDH and β‐Actin for target genes and SNORD68 and RNU6 for miRNA) and finally compared to the respective control sample.

### 2.6 Multiplex Immunoassay

To determine whether AOH impacts IL‐8, IL‐6, TNF‐α, and IL‐1β cytokine release, a multiplex immunoassay was conducted in IL‐1β stimulated cells. Applied AOH concentrations were chosen from the most interesting effects seen on transcript level. Caco‐2 cells were, hence, pre‐incubated for 2 h with AOH (0.2, 20, and 40 µm for short‐ and 0.02, 0.2, 20, and 40 µm for long‐term incubation) and subsequently, stimulated with IL‐1β (25 ng mL^−1^) for 3 or 18 h. The supernatants containing secreted cytokines were collected and centrifuged at 10 000 × *g* (4 °C) to separate secreted cytokines from cell residues. The cytokine concentration was determined using a ProcartaPlex Human Basic Kit and ProcartaPlex Human Simplex Kits (Affymetrix, Vienna, Austria) following the instructions of the manufacturer´s protocol. Briefly, a mixture of magnetic beads carrying antibodies for each cytokine were dispensed in a 96‐well plate and 50 µL of the respective supernatant was added into each well. After 2 h of incubation, a second cytokine dependent antibody was added and incubated for 30 min. The second antibody enabled the attachment of the fluorescent reporter streptavidin–phycoerythrin conjugate (SA‐PE), which finally allowed the quantification of secreted cytokines by measuring the fluorescence of the bound SA‐PE with the Luminex 200 System (BioRad, Vienna, Austria).

### 2.7 Statistical Analysis

Presented data are the mean ± SD of at least three independent biological replicates. Statistical significances were determined by using one‐way ANOVA via post hoc Holm–Bonferroni test and two sample *t‐*tests. The nonparametric Kruskal–Wallis ANOVA and Mann–Whitney *U* test were used to calculate statistical significances if normal distribution was not given. Values were considered as statistically different if *p* ≤ 0.05, ≤ 0.01, ≤ 0.001.

## 3 Results

### 3.1 Cell Viability

Possible cytotoxic properties of AOH on differentiated Caco‐2 cells were investigated with the alamarBlue assay after 5 and 20 h of exposure. Only a marginal, yet not significant, reduction in metabolic activity (92.38 ± 3.9%) by 20 µm AOH could be observed in non‐stimulated Caco‐2 monolayers after 5 h but was no longer prominent after 20 h of incubation. Overall, cell viability tests revealed that the tested AOH concentrations (0.02–40 µm) did not exhibit cytotoxic effects, neither in IL‐1β stimulated cells nor in non‐stimulated cells (data not shown).

### 3.2 AOH Alters Cytokine Gene Transcription in Differentiated Caco‐2 Cells

To investigate whether AOH itself modulates the transcription levels of the cytokine genes IL‐8, IL‐6, IL‐1β, and TNF‐α, differentiated Caco‐2 cells were incubated at concentrations of 0.02–40 µm for 5 and 20 h. In non‐stimulated Caco‐2 cells, the C_T_ values for IL‐6 and TNF‐α were too high to allow reproducible detection by qPCR (C_T_ ≥ 35.5, data not shown), irrespective of AOH incubation.

**Figure 2:**
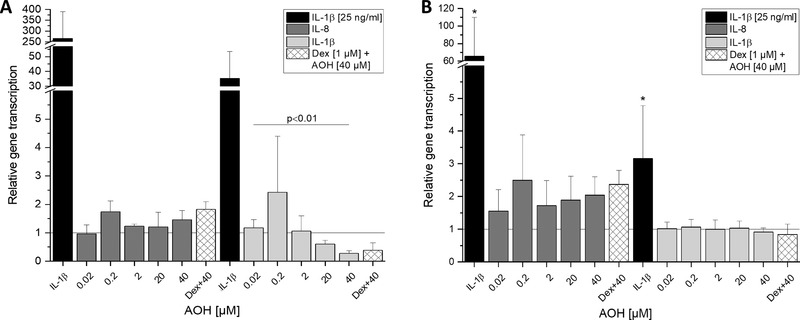
Impact of AOH on IL‐8 and IL‐1β cytokine gene transcription levels in differentiated Caco‐2 cells after 5 h (A) and 20 h (B) of incubation. IL‐1β (25 ng mL^−1^) is used as a positive control for proinflammatory effects and dexamethasone (Dex) as a control for potential anti‐inflammatory effects. Values are the mean ± SD of at least three independent experiments and presented as relative gene transcription (2^−ΔΔC^_T_) normalized to β‐actin and GAPDH and compared to the solvent control (1% DMSO, *y* = 1). Significant differences of the test concentrations were calculated by Kruskal–Wallis ANOVA and one‐way ANOVA for (A) and (B), respectively. Significances compared to the solvent control were calculated with Mann–Whitney *U* test (A) and a two‐sample *t*‐test (B; *p < 0.05).

IL‐1β (25 ng µL^−1^ IL‐1β) was used as a positive control to provoke an inflammatory stimulus in differentiated Caco‐2 cells. Observed differences in response to IL‐1β stimulation after 5 as well as after 20 h incubation (**Figure** 2) might result from the differentiation process, which is reasonable to affect the sensitivity to the stimulus.

In non‐stimulated cells, AOH did not show any modulatory effect on IL‐8 transcript levels after 5 h of incubation in comparison to the solvent control, but decreased IL‐1β transcript levels dose‐dependently (2–40 µm, Figure 2A). At the highest applied concentration (40 µm) of AOH, the transcript level of IL‐1β was potently reduced to 0.27 ± 0.09. After prolonging the incubation time to 20 h, however, the repressing effect of AOH on IL‐1β transcription was no longer pronounced (Figure 2B). For IL‐8, an inducing tendency was observed after 20 h for all test concentrations, however with no statistical significance. Also, for the co‐incubation of 40 µm AOH with dexamethasone (Dex) no significant difference was observed for any measured time point and gene.

### 3.3 AOH Modulates IL‐1β‐induced Cytokine Gene Transcription in Differentiated Caco‐2 Cells

**Figure 3:**
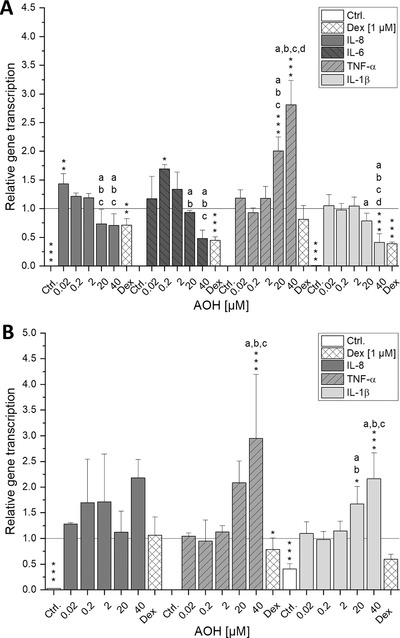
IL‐1β‐induced relative gene transcription levels of IL‐8, IL‐6, TNF‐α, and IL‐1β in differentiated Caco‐2 cells after AOH treatment. Caco‐2 monolayers were pre‐incubated for 2 h with AOH or Dex and additionally stimulated with IL‐1β (25 ng mL^−1^) for further 3 (A) or 18 h (B). Dexamethasone (Dex) is used as a potential positive control for anti‐inflammatory effects. Shown values are the mean ± SD of at least three independent experiments and presented as relative gene transcription (2^−ΔΔC^_T_) normalized to β‐actin and GAPDH and compared to IL‐1β stimulated cells (calibrator, *y* = 1). Significant differences among the test concentrations were calculated by one‐way ANOVA (*p* < 0.05, a–d). Statistical differences compared to the IL‐1β stimulus were calculated with a two‐sample *t*‐test (*p; **p; ***p < 0.05, 0.01, 0.001).

To study the possible immunomodulatory effect of AOH on IL‐1β‐induced cytokine gene transcription after short‐term and long‐term exposure (5 and 20 h), the transcript levels of IL‐8, IL‐6, IL‐1β, and TNF‐α in IL‐1β‐stimulated differentiated Caco‐2 cells were determined (**Figure** 3). The transcript level of the respective IL induced by IL‐1β (25 ng mL^−1^ IL‐1β and 1% DMSO) was set to 1 and served as calibrator for transcription analysis. After 2 h of pre‐incubation with AOH (0.02–40 µm) and subsequent co‐incubation for 3 h with IL‐1β (25 ng mL^−1^) a tendency to a drop in IL‐8, IL‐6, and IL‐1β transcripts was apparent at higher AOH concentrations (≥20 µm; Figure 3A). Interestingly, low test concentrations of AOH significantly increased IL‐8 (0.02 µm, *p* < 0.05) and IL‐6 (0.2 µm, *p *< 0.05) transcript levels when compared to solely IL‐1β stimulated cells (*y* = 1, Figure 3A), while the highest concentration of AOH rather reduced IL‐8 and IL‐6 transcription. However, the most striking observation emerging from the data was that TNF‐α showed a pronounced dose‐dependent and significant increase in gene transcription (20–40 µm AOH, *p* < 0.001). Especially 40 µm of AOH augmented the relative gene transcript level of TNF‐α 2.81 ± 0.43‐fold, compared to the IL‐1β‐stimulated control. The transcription of TNF‐α was significantly induced by high concentrations of AOH (≥20 µm) in contrast to the transcript levels of IL‐8, IL‐6, and IL‐1β, which were diminished. At a concentration of 40 µm AOH significantly reduced IL‐1β transcript levels to 0.41 ± 0.15 (*p* < 0.001). Furthermore, pre‐incubation with 1 µm Dex for 2 h resulted in a significant reduction of IL‐8 (*p* < 0.01), IL‐6, and IL‐1β (*p* < 0.001) mRNA, comparable to 40 µm AOH exposure. Solely, IL‐1β induced TNF‐α transcription could not be significantly reduced in the applied cell model by the corticosteroid Dex.

Analysis after 2 h of pre‐incubation with AOH (0.02–40 µm) and subsequent co‐incubation for 18 h with IL‐1β (25 ng mL^−1^) showed an inducing tendency for IL‐8 transcript levels (Figure 3B), while the effects could not be confirmed for IL‐6 gene transcription. As with unstimulated cells, the C_T_ values for IL‐6 were too high to obtain reproducible results (data not shown). Results for TNF‐α showed a similar transcription profile as observed after short‐term incubation, since the highest AOH concentration still significantly increased its transcript level (*p* < 0.001). In contrast to short‐term exposure, IL‐1β transcript levels were significantly increased at higher AOH test concentrations (20–40 µm, *p* < 0.05, 0.001).

### 3.4 Suppression of IL‐8 Secretion by AOH

**Figure 4:**
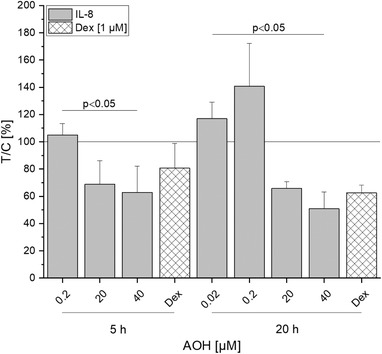
IL‐1β‐induced IL‐8 cytokine release of differentiated Caco‐2 cells after AOH treatment. Cell monolayers were pre‐incubated with AOH or Dex for 2 h and additionally stimulated with IL‐1β (25 ng mL^−1^) for further 3 or 18 h. Cells treated with Dex are used as a positive control for anti‐inflammatory effects. Values plotted are the mean ± SD of three independent experiments and presented as test over control in percentage (T/C (%)) and compared to IL‐1β stimulated cells (calibrator, *y* = 1). Significant differences of the test concentrations were calculated by Kruskal–Wallis ANOVA (*p* < 0.05). Statistical differences of cells treated with Dex compared to IL‐1β stimulated cells were calculated by the Mann–Whitney *U* test.

To study whether the modulatory effect of AOH on proinflammatory cytokine gene transcription is reflected at the cytokine secretion level, a magnetic bead‐based immunoassay was performed. Differentiated Caco‐2 cells were pre‐incubated for 2 h with AOH (0.02–40 µm) and subsequently co‐incubated for either 3 or 18 h with IL‐1β (25 ng mL^−1^). Preliminary experiments signaled that the suggested immunomodulatory response to AOH after stimulation with IL‐1β could only be demonstrated for IL‐8 at the secretion level (**Figure** 4). The response for IL‐6 and TNF‐α secretion was minor (data not shown). As the inflammatory response was induced with IL‐1β cytokine, IL‐1β secretion levels were, as expected, above the range of quantification. However, the results showed that already after 5 h of incubation IL‐8 secretion levels were significantly decreased by AOH (20–40 µm, *p* < 0.05) following a similar pattern as the transcription levels after short‐term incubation (Figure 3A). Further analysis of IL‐8 secretion after long‐term incubation (20 h) revealed a slightly increased secretion level at lower test concentrations, but again a suppression at higher AOH concentrations (20–40 µm). AOH at 40 µm even showed a slightly more pronounced inhibition of IL‐1β induced secretion than the anti‐inflammatory corticosteroid Dex at both time points, yet without statistical significance.

### 3.5 Impact of AOH on miRNA Transcript Levels

**Figure 5:**
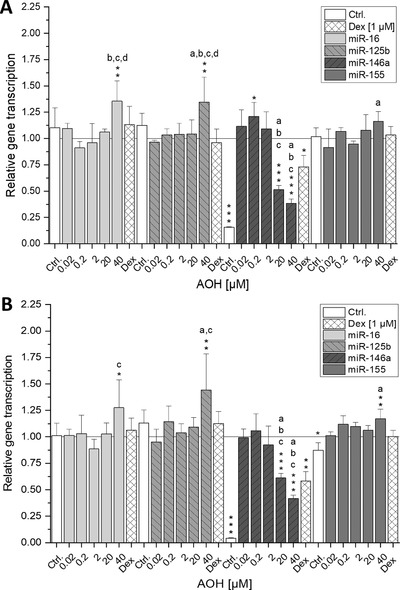
Impact of AOH on relative miRNA transcription levels of miR‐16, miR‐125b, miR‐146a, and miR‐155 in differentiated Caco‐2 cells. Caco‐2 monolayers were pre‐incubated for 2 h with AOH or Dex and additionally stimulated with IL‐1β (25 ng mL^−1^) for further 3 (A) or 18 h (B). Values are the mean ± SD of at least three independent experiments and presented as relative gene transcription (2^−ΔΔC^_T_) normalized to RNU6 and SNORD68 and compared to IL‐1β stimulated cells (calibrator, *y* = 1). Significant differences among the test concentrations were calculated by one‐way ANOVA (*p* < 0.05, a–d). Statistical differences compared to the IL‐1β stimulus were calculated with a two‐sample *t*‐test (*p; **p; ***p < 0.05, 0.01, 0.001).

To enlighten the mechanisms behind the potential anti‐inflammatory response to AOH observed at the cytokine transcription as well as at the secretion level, the impact of AOH on selected inflammation‐related miRNAs (miR‐16, miR‐125b, miR‐146a, and miR‐155) was investigated. These short RNAs are considered as important regulators of cytokine gene expression acting either as posttranscriptional regulators or as repressors of mRNA‐translation. To determine whether AOH modulates IL‐1β‐induced inflammatory response after 2 h of pre‐incubation and subsequent co‐incubation for 3 or 18 h with IL‐1β in differentiated Caco‐2 cells, qPCR was performed (**Figure** 5). The results showed significant induction of miR‐16 and miR‐125b by 40 µm of AOH after short‐term incubation (*p* < 0.01), whereas for miR‐155, only a minor inducing tendency was observed (Figure 5A). The most remarkable result was the significant suppression of miR‐146a transcript levels (*p* < 0.001) by 20 and 40 µm AOH. In contrast to that, 0.2 µm of AOH significantly increased miR‐146a levels (*p* < 0.05). Prolonged incubation (20 h) of differentiated Caco‐2 cells with AOH exhibited a similar transcription profile of the selected miRNAs to that of short‐term incubation (Figure 5B). Although the exposure to 0.2 µm AOH did no longer induce a significant increase of miR‐146a transcription, 20 and 40 µm of AOH still potently reduced IL‐1β induced miR‐146a levels (*p* < 0.001). The reduction observed with 40 µm of AOH was pronounced even more potently than with cells exposed to 1 µm Dex (*p* < 0.05). Furthermore, transcript levels of miR‐16, miR‐125b, and miR‐155 were still significantly increased after incubation with 40 µm of AOH (*p* < 0.05, 0.01, and 0.01, respectively).

## 4 Discussion

Intestinal epithelial cells (IECs) are, due to their location, easily exposed to high concentrations of food‐associated contaminants, such as mycotoxins. Surrounded by the rich network of the body's biggest accumulation of immune cells, IECs display crucial sentinels aiding to mount a proper innate immune response.^18^ The present work aimed to characterize the impact of AOH on inflammation‐related signaling at the gastrointestinal barrier, using differentiated Caco‐2 cells as a model. Corroborating with existing literature, where AOH, up to 100 µm, was found to be not cytotoxic after 24 h of incubation in non‐confluent Caco‐2 cells,^7^ no cytotoxic properties were observed in the applied concentration range (up to 40 µm AOH; data not shown). Without additional inflammatory stimulus, AOH displayed a tendency to induce IL‐8 transcription after long‐term incubation (20 h), albeit in a concentration‐independent manner (Figure 2). These findings are only in part supported by previous research, as AOH was reported to leave IL‐8 secretion unaffected in human primary macrophages^10^ or rather slightly reduce its transcription in THP‐1‐derived macrophages.^12^ This discrepancy might arise from differences between cell models of different tissue origin.

Possessing active UDP‐glucuronosyl‐ and sulfotransferases, Caco‐2 cells were reported to give rise to three metabolites (AOH‐3‐*O*‐sulfate and AOH‐9‐*O*‐ and AOH‐3‐*O*‐glucuronide).^21^ Since their properties are still unexplored, it cannot be excluded that soaring AOH metabolites might contribute to immunomodulatory effects after long‐term exposure, even though phase II metabolites are generally believed to exert less toxicity than their parent compound.^30^ The observed induction, however, is marginal compared to the reported potency of the known IL‐8‐inducing mycotoxin deoxynivalenol (DON) with 11‐fold IL‐8 transcript potentiation following 10 µm incubation.^25^

Existing literature suggested an immunosuppressive function of AOH, as it decreased the expression of several cell surface markers and TNF‐α transcription during the differentiation process of THP‐1 monocytes to macrophages.^11^ Since IL‐1β as well as TNF‐α represent early regulators of immunity, the observed significant reduction of transcript levels by AOH (20–40 µm) after 5 h (Figure 2A) suggests a dysregulation of steady IEC homeostasis that might further lead to suppressed immune function. To the best of our knowledge, this study is the first showing the effects of AOH on IL‐1β transcription in cells of the gastrointestinal tract.

The intestinal mucosa constantly exerts a condition of physiological inflammation, because it persistently faces myriads of bacterial or dietary antigens and a vast amount of other possibly mutagenic or toxic stimuli. During this state, not only immune cells but also endothelial, mesenchymal, nerve, and epithelial cells orchestrate the intestinal immune response and pathological or chronic inflammation might be induced by a dysregulation of any component of this highly sophisticated complex of intercellular signaling.^17^ Therefore, the potential impact of AOH on IL‐1β‐induced immune response was of interest, since an augmented secretion of the proinflammatory cytokine was reported during inflammation as well as chronic intestinal bowel diseases (IBDs).^31^

Corresponding well with recent literature, the extraneously provoked inflammatory response was repressed by AOH (20–40 µm). In more detail, the IL‐1β induced transcription of the proinflammatory cytokines IL‐8, IL‐6, and IL‐1β (Figure 3A) was inhibited after 5 h of incubation as well as the secretion of IL‐8 (Figure 4) was reduced after both 5 and 20 h of AOH exposure. Similar effects were reported for RAW 264.7 macrophages, BEAS lung epithelial cells, and THP‐1 cells, where AOH reduced LPS induced immune responses.^11^, ^12^, ^13^ As underlying mechanism, an inhibition of the LPS induced NF‐κB signaling pathway was reported in THP‐1 macrophages.^12^ Since stimulation of the IL‐1β receptor induces several signaling cascades including the activation of the NF‐κB transcription factor, an AOH mediated reduction of NF‐κB expression might explain the decreased response toward IL‐1β stimulation. However, this hypothesis does not apply for TNF‐α mRNA levels as they were significantly upregulated by 20–40 µm AOH at both time points, suggesting that TNF‐α transcription is mediated by a different pathway. Although a reducing tendency for IL‐8 and IL‐6 transcription by 40 µm could be observed, lower concentrations of AOH (0.02–0.2 µm) led to a significant induction of IL‐8 and IL‐6 transcription. Comparable results were obtained by Maresca et al., as low concentrations of the mycotoxins DON and patulin (25 µm) increased IL‐8 expression more potently than higher concentrations in Caco‐2 cells.^25^

Even though potently induced at the transcript level, protein secretion of TNF‐α was below LOD. Additionally, while long‐term exposure of stimulated Caco‐2 cells with AOH led to significant upregulation of IL‐1β and IL‐8 transcription (Figure 3B), IL‐8 protein secretion was still potently repressed by AOH (20–40 µm). In contrast, the regulated *Fusarium* mycotoxin DON (5 µg mL^–1^) was reported to show strong synergistic effects on IL‐8 secretion in IL‐1β stimulated Caco‐2 cells.^32^ In the acute phase of inflammation, IL‐8 secretion of IECs attracts and activates neutrophils.^33^ The inhibition of its secretion underlines the already reported immunosuppressive function of AOH, as a dysregulated immune response must be expected. Comparable results were reported for gliotoxin, a fungal metabolite that impeded not only NF‐κB activation but also TNF‐α and LPS induced IL‐8 production in colonic epithelial cells (SW620) and NR8383 rat macrophages, respectively.^34^

The observed inhibition of IL‐8 protein secretion after long‐term incubation with AOH occurred without concomitant reduction of the corresponding mRNA increase, likely being the result of a posttranscriptional regulatory process. Hence, in an attempt to go further in the understanding of the differentially affected transcriptional and translational level of the proinflammatory cytokine IL‐8 as well as the immeasurability of TNF‐α secretion, the influence of post‐transcriptionally interfering miRNAs was investigated. The measured inflammation related miRNAs miR‐146a, miR‐125b, miR‐16, and miR‐155 showed similar transcription patterns after both short‐ and long‐term exposure to AOH (Figure 5). Since only gene transcription of TNF‐α did not show opposing trends (Figure 3), the posttranscriptional interference of miRNAs seemed likely. Indeed, miR‐16 and miR‐125b—which were significantly increased by 40 µm AOH—were reported to mediate TNF‐α destabilization through interaction with tristetrapolin, an RNA‐binding protein targeting cytokine encoding mRNAs at their AU‐rich elements and direct binding to its 3′ untranslated region, respectively. Although the observed slight induction of miR‐155 (40 µm AOH) is rather thought to stabilize TNF‐α mRNA, other predicted targets of this miRNA include several important mediators of the IL‐1β signaling pathway. Hence, miR‐155 overexpression might inhibit the activation of the signaling cascade and thereby suppress cytokine expression.^15^, ^35^ In line with recent literature, 20–40 µm AOH significantly suppressed transcription of the NF‐κB dependent miR‐146a.^12^ Besides miR‐146a being reported to negatively correlate to TNF‐α transcription, two predicted targets of miR‐146a (TRAF‐6 and IRAK‐1) are crucial mediators in the upstream of the NF‐κB signaling cascade.^15^, ^36^, ^37^ Thus, it can be presumed that the reduction of miR‐146a transcription counteracts AOH‐mediated inhibition of NF‐κB activation.

To date, data on the occurrence and hazard characterization of AOH are still limited. But AOH has been reported at low µg kg^–1^ levels in a broad variety of food and feed. For instance, in a recent food survey AOH was measured in sunflower seed oil, wheat flour and tomato sauce at median concentrations of 1.2, 2.1, and 6.6 µg kg^–1^, respectively.^38^ With regard to the estimated daily dietary intake of 1.0–15.2 ng kg^–1^ bw^3^ the observed inducing effects of AOH on IL‐8 transcription and secretion at concentrations of 0.02 and 0.2 µm, respectively, need to be further investigated. These concentrations can be considered as physiologically relevant and immuno‐stimulating properties of AOH have not been described yet. Contamination of single food products with higher amounts of AOH, however, cannot be precluded as no monitoring or regulatory parameters exist so far. In carob fruit for instance 180 µg kg^–1^ AOH were detected whereas in sorghum‐based mixed feed a maximum value of 1200 µg kg^–1^ AOH could be found.^2^, ^3^ These data underline the necessity of thorough exposure assessment and to take immunomodulatory properties into consideration for respective hazard characterization.

Furthermore, combinatory effects with other immunomodulating food constituents, e.g., the anthocyanin cyanidin 3‐glucoside (Cy3glc), a plant pigment ubiquitously occurring in berries and plums at low mg kg^–1^ levels,^39^, ^40^ might be taken into account, since AOH was also reported to contaminate these edibles.^3^ Cy3glc, like AOH, was reported to have immunosuppressive properties at comparable concentrations (20–40 µm) in Caco‐2 cells. For instance, Cy3glc reduced the TNF‐α‐induced activation of NF‐κB and transcription of IL‐6, IL‐8, and TNF‐α.^41^, ^42^

Taken together, the present work provides the first evidence of the emerging mycotoxin AOH exerting immunomodulatory properties in non‐immune cells of the intestinal epithelium. Following IL‐1β stimulation, the immunosuppressive impact of AOH (20–40 µm) on IL‐8, IL‐6, and IL‐1β transcription as well as IL‐8 secretion could be demonstrated, indicating that this emerging mycotoxin might not only manipulate LPS‐ but also IL‐1β‐related pathways. Furthermore, AOH affected regulatory miRNAs that are possibly targeting cytokines for posttranscriptional destabilization or interfering with signaling pathway molecules. At present, the relevance of systemic immunomodulatory effects of AOH in the higher micromolar range remains to be clarified with respect to exposure levels and bioavailability. However, the present study demonstrates that AOH might affect the immune response of cells at the gastrointestinal barrier.

## Conflict of Interest

The authors declare no conflict of interest.
